# Neural correlates of the DMT experience assessed with multivariate EEG

**DOI:** 10.1038/s41598-019-51974-4

**Published:** 2019-11-19

**Authors:** Christopher Timmermann, Leor Roseman, Michael Schartner, Raphael Milliere, Luke T. J. Williams, David Erritzoe, Suresh Muthukumaraswamy, Michael Ashton, Adam Bendrioua, Okdeep Kaur, Samuel Turton, Matthew M. Nour, Camilla M. Day, Robert Leech, David J. Nutt, Robin L. Carhart-Harris

**Affiliations:** 1 Centre for Psychedelic Research, Department of Brain Sciences, Faculty of Medicine, Imperial College, London, UK; 2 Computational, Cognitive and Clinical Neuroscience Laboratory (C3NL), Faculty of Medicine, Imperial College, London, UK; 3 Department of Basic Neurosciences, University of Geneva, Geneva, Switzerland; 4 Faculty of Philosophy, University of Oxford, Oxford, United Kingdom; 5 School of Pharmacy, The University of Auckland, Auckland, New Zealand; 6 PKDM Unit, Department of Pharmacology, University of Gothenburg, Gothenburg, Sweden; 7 Imperial Clinical Research Facility, Imperial College London, London, UK; 8 Centre for Psychiatry, Division of Brain Sciences, Department of Medicine, Imperial College, London, UK; 9 Department of Psychosis Studies, Institute of Psychiatry, Psychology and Neuroscience, King’s College London, London, UK; 10 Department of Neuroimaging, Institute of Psychiatry, Psychology and Neuroscience, King’s College London, London, UK; 11 Department of Psychiatry, University of Oxford, Oxford, UK

**Keywords:** Consciousness, Perception, Human behaviour

## Abstract

Studying transitions in and out of the altered state of consciousness caused by intravenous (IV) N,N-Dimethyltryptamine (DMT - a fast-acting tryptamine psychedelic) offers a safe and powerful means of advancing knowledge on the neurobiology of conscious states. Here we sought to investigate the effects of IV DMT on the power spectrum and signal diversity of human brain activity (6 female, 7 male) recorded via multivariate EEG, and plot relationships between subjective experience, brain activity and drug plasma concentrations across time. Compared with placebo, DMT markedly reduced oscillatory power in the *alpha* and *beta* bands and robustly increased spontaneous signal diversity. Time-referenced and neurophenomenological analyses revealed close relationships between changes in various aspects of subjective experience and changes in brain activity. Importantly, the emergence of oscillatory activity within the delta and theta frequency bands was found to correlate with the peak of the experience - particularly its eyes-closed visual component. These findings highlight marked changes in oscillatory activity and signal diversity with DMT that parallel broad and specific components of the subjective experience, thus advancing our understanding of the neurobiological underpinnings of immersive states of consciousness.

## Introduction

N, N, Dimethyltryptamine (DMT) is a naturally-occurring serotonergic psychedelic^1^ capable of producing experiences that, in intensity, surpass those associated with standard doses of most orally administered psychedelics and indeed most other categories of psychoactive drugs^2,3^. The subjective effects of intravenous DMT have a rapid onset and are characterized by unusually vivid visual imagery and somatic effects, which arise within seconds of the injection. At high doses, the experience rapidly progresses into a deep and profound immersion - sometimes described as a ‘breakthrough’. This experience is often characterized by a sense of entering into an entirely ‘*other*’ but no less ‘*real*’ world or dimension^3,4^. It is not uncommon for people to describe encounters with sentient ‘entities’ or ‘presences’ within this perceived other world^4,5^ and for the experience to subsequently challenge beliefs about the nature of reality and consciousness.

The phenomenology of the DMT experience suggests it may be an especially powerful scientific tool for illuminating the neurobiology of consciousness. DMT experiences can be said to resemble ‘world-analogue’ experiences (i.e. interior analogues of external worlds) – similar to the dream state^6^. It is logical to presume that conscious processing becomes ‘functionally deafferented’ (i.e. cut-off) from the external sensorium in these states, paralleled by what is presumably an entirely internally generated ‘simulation state’, felt as entry into an entirely other world^5^. The rapid, short-acting and dramatic subjective effects of intravenous DMT therefore render it well-suited for investigating the neurobiology of consciousness with functional brain imaging – and this is what we sought to exploit here.

Previous EEG and magnetoencephalography (MEG) studies of psychedelic-induced changes in brain activity have yielded generally consistent results. While broadband decreases in (absolute) oscillatory power have been seen with psilocybin^7^, LSD^8^, and (the DMT-containing ceremonial brew) ayahuasca^9^, some evidence suggest that these reductions are more pronounced in certain frequency bands^10,11^, e.g. suppression of *alpha* power is perhaps the most robust and reliable functional brain effect of psychedelics. Supplementing traditional frequency-based analyses, another reliable finding is increased complexity, diversity, or entropy of brain activity under psychedelics^12^ – an intriguing result given that related measures are known to reliably index a range of different conscious states^13–15^.

Recent advances in analytical methods have enabled the decomposition of spectral power into its oscillatory and fractal (1/f) components^16^ and these components are thought to have distinct functional relevancies^17^. Importantly, oscillatory activity has been linked to specific generative mechanisms. For example, alpha power has been sourced to thalamic and visual cortical generators and a top-down suppressive function^18^, while theta power has been associated with limbic generators^19^ and learning^20^. Conversely, the origin and functional relevance of 1/f activity is poorly understood^17,21,22^. Psychedelics have, however, been found to affect 1/f-related activity^21,23^ – and this has also been seen in other atypical states of consciousness^16^. For these reasons, we assessed the effects DMT on rhythmic and fractal activity both separately and in combination in order to examine whether they are differently affected by DMT and how such changes relate to the different aspects of the DMT experience. EEG was chosen as a preferred brain imaging modality (i.e. over fMRI) due to its high temporal resolution, related ability to capture the fast-acting effects of DMT, and the known effects of psychedelics on vascular activity^1^ - which may be confounded with neuronal activity when using functional Magnetic Resonance Imaging; fMRI^24^.

The primary aim of our study was to determine how a bolus intravenous injection of DMT (versus a bolus intravenous injection of saline) affects the power spectrum and signal diversity of EEG recorded brain activity. Further, we aimed to establish the relationship between these brain activity measures, the real-time progression of the subjective experience and parallel changes in plasma levels of DMT. Finally, both conventional psychometric analyses and more temporally finessed methods - inspired by neurophenomenology^25^ - were utilized to assist the process of mapping between brain and experience. Subjective reports were obtained both via questionnaires and micro-phenomenological interviews, which promote detailed chronological accounts of subjective experiences, while reducing biases commonly associated with first person reports^26,27^ - making this technique ideal for neurophenomenological analyses. Our primary hypothesis was that DMT would decrease oscillatory power in the *alpha* band and increase cortical signal diversity and that these effects would correlate with changes in conscious experience across time.

## Results

### Subjective effects

**Figure 1:**
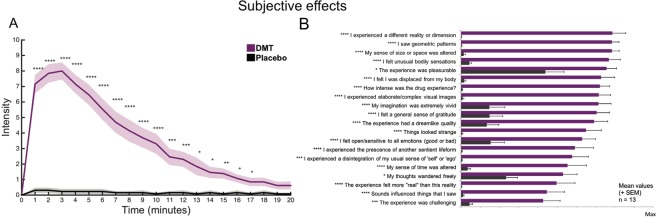
Subjective effects. (**A**) Real-time intensity ratings for DMT and placebo (mean ± SEM) (****p < 0.001; ***p < 0.005; **p < 0.01; * p < 0.05, FDR corrected, N = 13). (**B**) Visual analogue scales depict the phenomenological features of DMT and placebo (mean + SEM) (****p < 0.001; ***p < 0.005; **p < 0.01; *p < 0.05, FDR corrected, N = 13).

Participants were asked to provide ratings of the subjective intensity of the drug effects at every minute for a total of 20 minutes after DMT and placebo administration. Figure 1A displays the group-averaged intensity plots for each minute for a total of 20 minutes post-injection. Paired T-tests revealed that the subjective intensity of the experience remained significantly higher under DMT vs placebo for 17 minutes post-dosing (False Discovery Rate - FDR corrected) and peak subjective effects occurred 2–3 minutes post-injection.

Supplementing these basic intensity ratings, participants were asked to rate different aspects of their experiences using various visual analogue scales (VAS). All items were rated significantly higher in the DMT condition compared with placebo (FDR corrected) **(**Fig. 1B). Ratings were given retrospectively, at ~30 minutes following administration, i.e. once the acute effects of DMT had sufficiently subsided.

### Time-averaged EEG results

**Figure 2:**
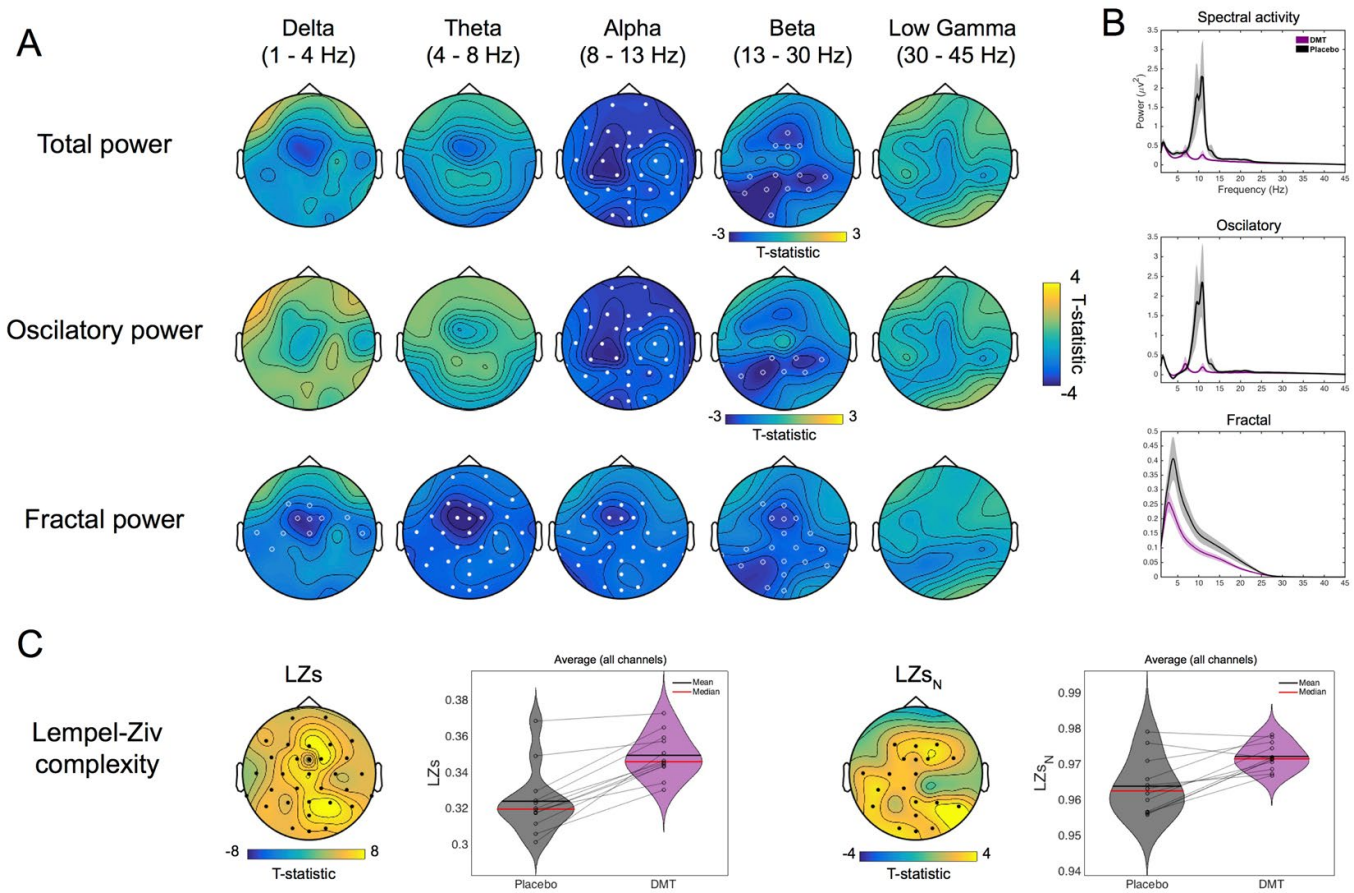
Time-averaged EEG results. (**A**) The comparison of DMT versus placebo for changes in spectral activity reveals significant decreases for the *alpha* and *beta* bands for conventional spectral power. The decomposed spectra into oscillatory and fractal power, revealed similar results for the former and reductions were seen on all bands < 30 Hz for the latter. Filled circles correspond to clusters p < 0.01 and hollow circles for clusters p < 0.05, N = 12. (**B**) Grand-average spectral power for DMT and placebo corresponding to spectral, oscillatory and fractal (1/f) components of the signal (N = 12). (**C**) Increases are seen for both measures of spontaneous signal diversity following DMT administration compared to placebo Filled circles correspond to clusters p < 0.01 and hollow circles for clusters p < 0.05, N = 12 (LZs = Lempel-Ziv complexity, LZs_N_ = normalized LZs).

Averaged EEG from the first 5 minutes of resting state activity following administration of DMT was contrasted against the same period after placebo, focusing on changes in the power spectrum and spontaneous signal diversity (LZs and LZs_N_). One participant was excluded due to excessive movement artifacts following DMT. Supporting one aspect of our primary hypothesis, contrasts revealed spatially-widespread and statistically-marked decreases in the *alpha band* (max. t(11) = −3.87, cluster p = 5.33e-04) and more modest decreases in the *beta* band (max. t(11) = −3.30, cluster p = 0.033) under DMT (Fig. 2A).

Decomposing the EEG spectra into its oscillatory and fractal (1/f) components revealed consistent changes in oscillatory power as reported above (*alpha*: max. t(11) = −3.87, cluster p = 2.67e-04. *Beta*: max. t(11) = −3.11, cluster p = 0.04), while the fractal component showed significantly reduced power within all frequency bands < 30 Hz (*delta*: max. t(11) = −3.91, cluster p = 0.024. *Theta*: max. t(11) = −4.28, cluster p = 0.007. *Alpha*: max. t(11) = −3.67, cluster p = 0.014. *Beta*: max. t(11) = −3.48, cluster p = 0.02) (Fig. 2A and see Fig. S1 for detailed results for DMT and placebo separately).

Signal diversity, as measured by Lempel-Ziv complexity (LZs) and normalized Lempel-Ziv complexity (LZs_N_), was significantly increased under DMT relative to placebo (max. t(11) = 8.09, cluster p = 2.67e-04; max. t(11) = 4.26, cluster p = 0.0029), respectively (Fig. 2C) – consistent with our primary hypothesis.

Results also revealed the emergence of prominent *theta* oscillations under DMT, to the extent that this rhythm replaced alpha as the peak frequency in terms of power (within the 4–45 Hz range: mean = 7.36 Hz, SEM = 0.68). This emergent theta rhythm under DMT was more evident in the oscillatory power spectrum, i.e. once the fractal component had been removed. As expected, an *alpha* peak was maintained throughout the placebo session (mean = 9.28 Hz, SEM = 0.62) (t(11) = −2.52, p = 0.029) (Fig. 2B).

### Time-sensitive EEG results

In addition to consistent alpha and *beta* reductions, minute-by-minute analyses revealed decreases in *delta* and *theta* bands only for the first minute post DMT injection – after which recovery (and increases in *theta* for the oscillatory component) were identified at minutes 2–3. These results indicate that DMT induces a general decrease in total power across all frequency bands between 1 and 30 Hz; however, there is a transient normalization/increases in *theta and delta* frequencies at the time of peak subjective intensity, which is especially evident in the oscillatory component of the signal. The spontaneous signal diversity measure, LZs, was found to be consistently increased for the whole of the post-injection period - and increases in LZs_N_ were evident from the time of peak intensity onwards (Fig. S2).

### Subjective vs EEG effects across time

**Figure 3:**
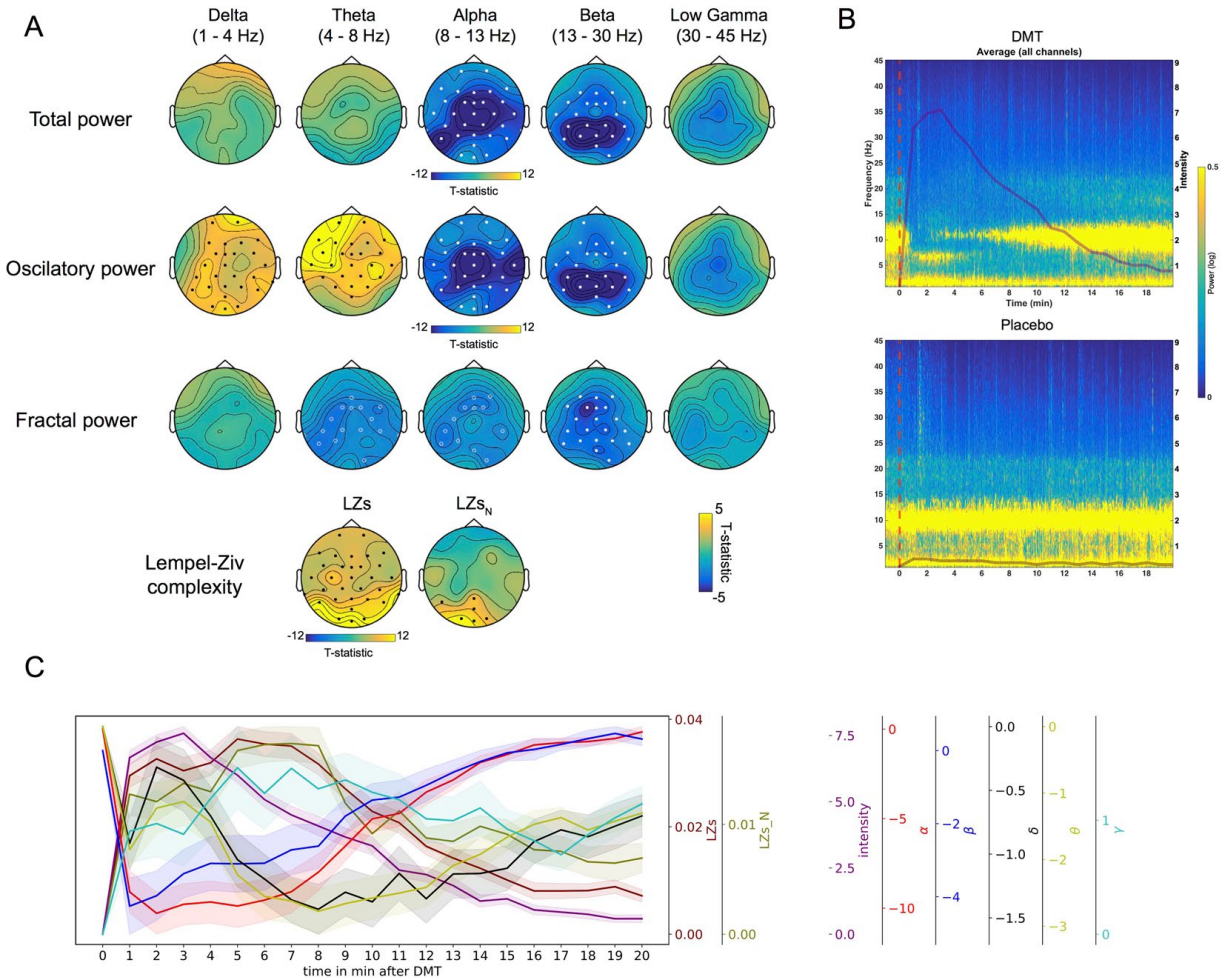
Subjective vs EEG effects across time. (**A**) Significant inverse relationships were found between real-time intensity ratings and power in *alpha* and *beta* bands for all power measures (including the *theta* band for fractal power). A positive relationship was found between intensity and power at *delta* and *theta* bands in the oscillatory component. Increased signal diversity (LZs and LZs_N_) correlated positively with intensity ratings also. Filled circles/dots correspond to clusters p < 0.01 and hollow circles for clusters p < 0.05, N = 12. Positive relationships are shown using black dots and negative relationships are shown using white dots/circles (**B**) Time frequency plot illustrating the associations between intensity ratings and spectral activity for DMT and placebo (red line marks beginning of injection), N = 12. (**C**) Temporal development of intensity, and EEG measures of spectral activity and spontaneous signal diversity (mean ± SEM, N = 12). (δ = *delta*, θ = *theta*, γ = *gamma*, α = *alpha*, β = *beta*, LZs = Lempel-Ziv complexity, LZs_N_ = normalized LZs).

In order to assess the relationship between the subjective and EEG changes across time, data was segmented into one-minute blocks. Results revealed a negative correlation between changes in total *alpha* power under DMT and ratings of subjective *intensity* that was significant for all recorded channels (max. t(11) = −16.05, cluster p = 2.67e-04; white dots, Fig. 3A). Decreased *beta* power similarly correlated with higher intensity ratings (max. t(11) = −9.83, cluster p = 0.0043). Analysis performed on just the oscillatory component of the signal showed mostly consistent results (*alpha*: max. t(11) = −14.85, cluster p = 2.67e-04. *Beta*: max. t(11) = −11.08, cluster p = 0.004), although additional positive correlations between *intensity* and *delta* (max. t(11) = 4.68, cluster p = 0.007) and *theta* power (max. t(11) = 7.17, cluster p = 0.003) also emerged as statistically significant (black dots, Fig. 3A). Fractal power revealed a negative correlation between (higher) intensity ratings and (reduced) *theta* (max. t(11) = −3.13, cluster p = 0.04), *alpha* (max. t(11) = −3.12, cluster p = 0.047) and *beta* power (max. t(11) = −5.21, cluster p = 0.01). These results reveal the emergence of a functionally relevant rhythmicity within the delta and theta frequency bands under DMT that was not evident in total power, which includes the fractal component of the signal. In fact, within the theta band, the fractal component appears to behave in an opposite way to the oscillatory component under DMT, i.e. there is increased theta power in the oscillatory component but decreased theta in the fractal component.

Supporting our primary hypothesis, increased signal diversity (LZs) under DMT correlated positively with subjective *intensity* ratings in posterior and central channels (max. t(11) = 9.96, cluster p = 2.67e-04). After controlling for changes in spectral power (LZs_N_), the relationship between signal diversity and *intensity* remained positive in the posterior channels only (max. t(11) = 5.11, cluster p = 0.009).

### Plasma DMT vs EEG effects

**Figure 4:**
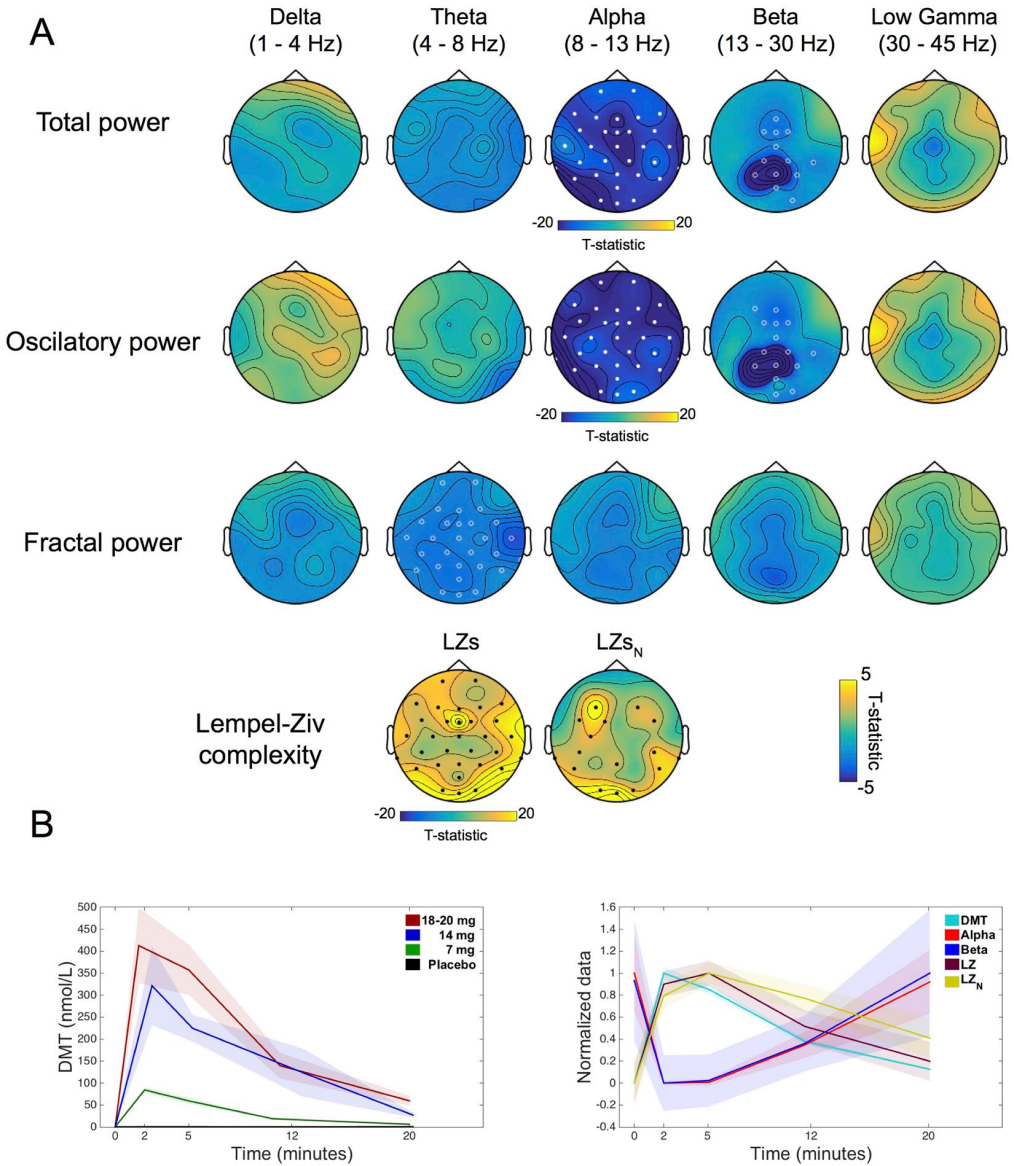
Plasma DMT vs EEG effects. (**A**) Significant inverse relationships (white dots/circles) were found between plasma levels of DMT and power in the *alpha* and *beta* bands for spectral and oscillatory power, while the relationship was found for plasma DMT and power in the *theta* band for fractal power. A positive relationship (black dots) was found between plasma levels of DMT and complexity measures (LZs and LZs_N_). Filled circles correspond to clusters p < 0.01 and hollow circles for clusters p < 0.05, N = 12. (**B**) Temporal development of DMT plasma concentrations, and EEG measures of total power and spontaneous signal diversity which were found significant to have a significant effect (mean ± SEM, N = 12).

Here we assessed the relationship between changes in the EEG data (frequency bands and signal diversity) and plasma concentrations of DMT across time. In a similar manner to the subjective values, higher concentrations of DMT in the blood were associated with greater reductions in *alpha* (max. t(11) = −23.59, cluster p = 2.67e-04) and *beta* power (max. t(11) = −12.61, cluster p = 0.04). Similar effects were seen when analyses were performed on the oscillatory component of the signal (*alpha*: max. t(11) = −28.32, cluster p = 2.67e-04. *Beta*: max. t(11) = −20.62, cluster p = 0.022), while the fractal component showed negative correlation between (higher) plasma concentrations and (reduced) *theta* power (max. t(11) = −4.11, cluster p = 0.038). As predicted, a relationship was also evident between plasma DMT and increased signal diversity (LZs: max. t(11) = 25.61, cluster p = 2.67e-04 and LZs normalized: max. t(11) = 7.63, cluster p = 2.67e-04) (Fig. 4).

### Neurophenomenology

**Figure 5:**
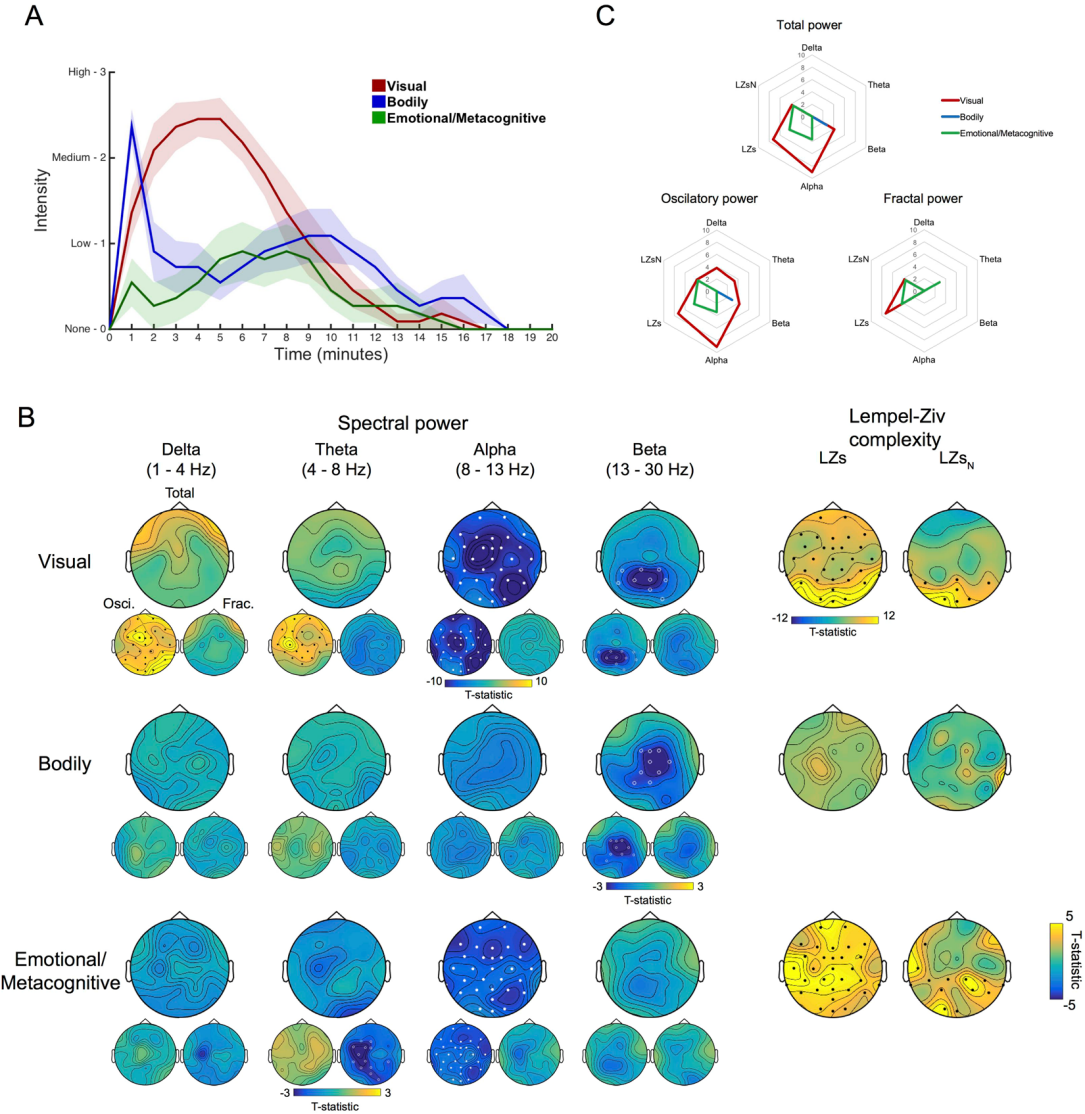
Neurophenomenology. (**A**) Average ratings (mean ± SEM) regarding the intensity for the three dimensions of experience which were found to be commonly altered across all participants following DMT administration. (**B**) Significant inverse relationships (white dots/circles) were found between the progression of visual effects induced by DMT and power at the *alpha* and the *beta* bands, as well as increases (black dots) in complexity (LZs and LZs_N_). Decreases of central *beta* band power showed a significant association the trajectory of bodily effects. Decreases in *alpha* band power and increases in complexity (LZs and LZs_N_) were significantly associated to the dynamics of emotional/metacognitive effects. Mostly consistent results were found with oscillatory power, however an intriguing positive relationship found with power at *delta* and *theta* bands for visual effects and reduced *theta* activity was linked to emotional/metacognitive effects. Filled circles/dots correspond to clusters p < 0.01 and hollow circles for clusters p < 0.05, N = 11. (**C**) Radar plots displaying the constellation of EEG effects associated to different dimensions of experience (mean values displayed).

Micro-phenomenological interviews (MPIs)^26,28,29^ were performed post-hoc to discover distinct (core) components of the subjective experience – that could then be used to constrain participant ratings referenced to specific time points, i.e. each passing minute. Three major common dimensions were found across participants, i.e.: (1) visual, (2) bodily and (3) emotional/metacognitive experiences. These dimensions were extracted from each of the participants’ interviews and then rated by a researcher not involved in EEG analysis with reference to each passing minute within the 20-minute recording period (see Methods for details) (Fig. 5A).

Adopting a data-led approach, we chose to look at components of the EEG that had already shown interesting relationships with intensity ratings, namely: *alpha*, *beta*, *delta* and *theta* power, plus signal diversity measures (LZs and LZs_N_). Results revealed a negative correlation between changes in visual intensity and changes in total *alpha* (max. t(11) = −14.24, cluster p = 2.67e-04) and *beta* (max. t(11) = −6.17, cluster p = 0.04) power as well as a positive relationship with changes in *delta* (max. t(11) = 6.06, cluster p = 0.004) and *theta* (max. t(11) = 6.59, cluster p = 0.01) power - when just the oscillatory component of the signal was used for analyses. Similar positive relationships between intensity ratings and EEG measures were seen for LZ (i.e. LZs: max. t(11) = 16.74, cluster p = 2.67e-04 and LZs_N_: max. t(11) = 6.62, cluster p = 0.008). Ratings of bodily effects were negatively correlated with changes in the *beta* band (max. t(11) = 3.17, cluster p = 0.0496) only. Lastly changes in the emotional/metacognitive dimension were primarily associated with decreases in the *alpha* band (max. t(11) = −4.56, cluster p = 0.008) and increases in the LZ measures (LZs: max. t(11) = 6.15, cluster p = 0.003 and LZs_N_: max. t(11) = 5.39, cluster p = 2.67e-04) (Fig. 5B). These results support and extend on our primary hypothesis.

### Psychometric correlational analyses

**Figure 6:**
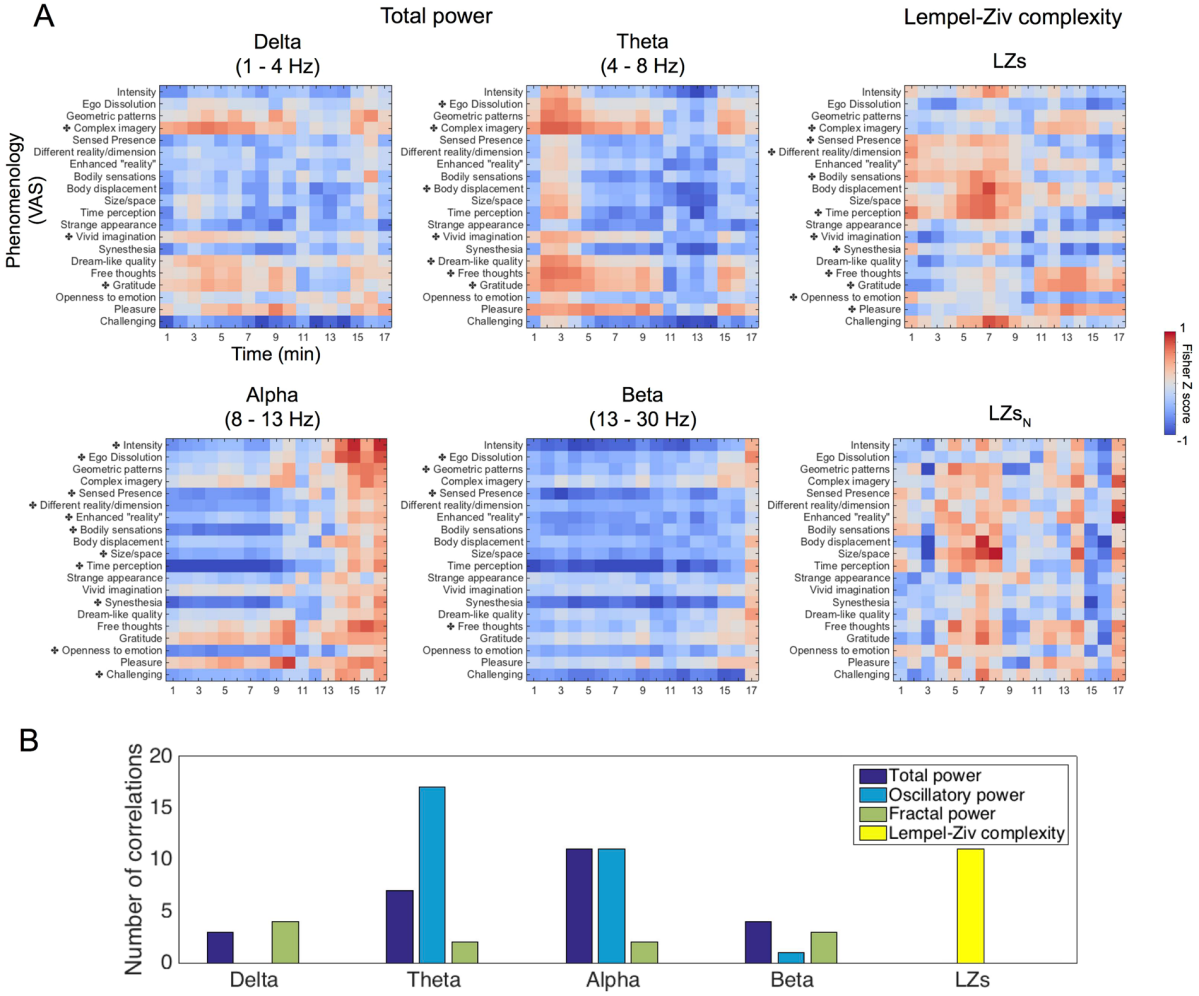
Psychometric correlational analyses. (**A**) Normalized correlation coefficient values between VAS items and EEG measures for each minute following DMT. Significant correlations between these normalized correlation values and intensity ratings are marked with a cross following Bonferroni-correction for multiple comparisons at p < 0.05 (see Fig. S3 for correlations with oscillatory and fractal power and Fig. S4 for 5-minute averaged data correlations). (**B**) Bar chart displaying the number of significant correlations between normalized correlation values (EEG metrics vs VAS items) and intensity ratings. Results revealed that *alpha* correlate most with subjective experience when the total power is assessed, whereas *theta* correlates most when just the oscillatory power is extracted. Conversely, fractal power displayed a small amount of significant correlations (See Fig. S6 for the specific correlation values for each item for oscillatory and fractal power). Finally LZs was a metric which displayed the same amount of significant correlations as *alpha* in total and oscillatory power.

In order to further test the relationship between specific EEG measures and the DMT experience, we performed additional post-hoc ‘subjective rating vs EEG’ correlations using both time-sensitive (minute-by-minute) and time-averaged analyses. Time-sensitive analysis revealed relationships that were broadly consistent with those reported above, i.e. negative correlations were evident between (higher) VAS item scores and (reduced) *alpha/beta* power and positive correlations were evident between (higher) VAS scores and (increased) LZs/*theta/delta* - during the period of peak subjective intensity (Fig. 6A and see Fig. S3 for specific correlations when oscillatory and fractal components of the signal were used).

Lastly, as one might expect, when group-averaged intensity ratings were correlated with Fisher’s Z normalized coefficient scores across time (i.e. minute-by-minute correlational coefficients for VAS item scores vs the relevant EEG-based values) – the subsequent relationships closely resembled those highlighted by the neurophenomenological analyses. Specifically, changes in *alpha* correlated strongly with the subjective experience when total and oscillatory power was used, while *theta* changes correlated strongly when only the oscillatory component was employed for analysis. Also, after the fractal component was extracted correlations with spectral data were overall reduced, i.e. the fractal component appeared to be less functionally relevant than the oscillatory component. Finally the amount of correlations between LZs and subjective ratings were found to be comparable to the number found when *alpha* power was used (Fig. 6B) (see Fig. S4 for time-averaged psychometric correlations).

## Discussion

This paper presents results from the first ever placebo-controlled investigation of the effects of DMT on spontaneous human brain activity. Immersion into the DMT state was accompanied by marked decreases in total spectral power in *alpha* and *beta* bands paralleled by marked increases in spontaneous signal diversity and the emergence of *theta* and *delta* oscillations during peak effects. These effects correlated significantly with the characteristic visual effects of DMT and represent novel discoveries for psychedelic neuroscience. The increases in *delta* and *theta* oscillations were most clearly evident when the oscillatory component was separated from the fractal component, suggesting that the former is the more functionally relevant component of the signal - at least in relation to these lower (EEG-recordable) frequency bands.

Decreased *alpha* power is a particularly consistent finding in neuroimaging research with psychedelics^7,8,11^. Alpha is the most prominent rhythm of the resting-brain, particularly in humans, and particularly in adulthood^30^. *Alpha* has been linked with high-level psychological functioning^31,32^, top-down predictive processing^18,33^ and related feedback connectivity^34^ - all of which have been found to be disrupted under serotonergic psychedelics^35–37^. Serotonin 2 A receptor antagonist (ketanserin) pretreatment studies involving both psilocybin^38^ and ayahuasca^39^ have supported the principle that psychedelic-induced reductions in *alpha* power depend on activation of 5-HT2A receptors. Here we found strong correlations between *alpha* power decreases, minute-by-minute changes in the subjective intensity, and DMT levels in plasma.

The present study’s findings of profound *alpha* suppression, combined with normalized/increased *delta* and *theta* under DMT may relate to the experience of feeling profoundly immersed in an entirely other world. The emergence of *theta*/*delta* oscillations, particularly in medial temporal lobe sources, has been classically associated with REM sleep dreaming and related ‘visionary’ states^40,41^. We propose that the observed emergence of *theta/delta* rhythmicity combined with the characteristic ‘collapse’ of *alpha/beta* rhythmicity under DMT may relate to the ‘DMT breakthrough experience’ – a perceptual mechanism by which the brain switches from the processing of exogenously incoming information to a state in which processing is endogenously-driven, as in classic REM sleep dreaming^6^. This is further supported by the observed positive correlation between participants’ ratings of the visual quality of their experiences and increases in *theta* and *delta* power – as well as decreases in *alpha*. Although speculative, it is intriguing to consider that the emergent *theta/delta* rhythmicity under DMT (also observed in a non-controlled field study^42^) may have a deep (e.g. medial temporal lobe) source and reflect the recruitment of an evolutionarily ancient circuitry that has been classically associated with REM-sleep and medial temporal lobe stimulation – both of which are known to feature complex visionary phenomena^40^.

The increases in signal diversity found here, as elsewhere^12^ may be considered the positive complement of reduced *alpha* power and are consistent with the so-called ‘entropic brain hypothesis’ which proposes that within a limited range of states (i.e. within a critical zone) the richness of content of any given conscious state, can be meaningful indexed by the entropy of its spontaneous brain activity^13,14^. Based on the present study’s findings of a strong and comprehensive relationship between spontaneous signal diversity (a measure intimately related to entropy^43^) and the temporal evolution of different aspects of DMT’s subjective effects, we maintain that entropy-related measures are indeed informative indices of the quality of a given state of consciousness^13,14^. Using a variety of imaging metrics and drugs, an increasing number of studies have reported increased signal complexity, diversity or entropy under psychedelics^15,44,45^. The increases in signal diversity observed here were associated with the perceived intensity of the experience, levels of DMT in plasma and a range of subjective effects. The increases in signal diversity were inversely with *alpha* power but had a different EEG topography – i.e. increased LZs was most pronounced in occipital electrodes, whereas alpha reductions were strongest in central channels. These findings further corroborate the view that increased LZs is related to the stark visual quality of the DMT experience (see Figs 3A and 5B) and is an informative complement to traditional spectral power analyses – particularly when investigating psychedelic states.

The neurophenomenological approach and psychometric correlations employed here, using real-time measures and micro-phenomenological interviews, represent a positive step towards the integration of neuronal and first-person reports^25,46^. The relevant analyses and results allowed us to establish robust and specific relationships between subjective effects – i.e. in the visual, somatic and metacognitive/affective domains - and different aspects of the EEG data. Combining multimodal brain imaging with such advancements in subjective data analysis may further aid our understanding of the neural correlates of the psychedelic experience – and indeed other interesting conscious states.

Finally, the present results may shed light on the mechanisms underpinning the antidepressant potential of DMT and DMT-related compounds^47,48^. Increased *alpha* power and decreased *delta* power has been found in populations of depressed individuals^49^ and associations have been observed between signal diversity and fluctuations in mood^50^ including depressive states^51^. It is reasonable to consider that the massive effects observed here under DMT may have implications for modelling, and perhaps treating, psychopathology.

Addressing some limitations: For safety-related, dose-finding reasons, four different doses of DMT were administered to participants. This created non-uniformity between the participants’ experiences but likely aided correlational analyses. The fixed-order design could be considered a limitation. However, previous psychedelic neuroimaging work of ours using fixed and balanced order designs^7^ have yielded consistent results with those seen here. Moreover, fixed order designs circumvent the issue of problematic carry-over effects - which are likely given the enduring psychological effects of psychedelics^52^.

To conclude, this is the first report on the resting-state brain effects of intravenous DMT in humans. EEG recordings revealed decreased spectral power in the *alpha*/*beta* bands, accompanied by widespread increases in signal diversity. The temporal dynamics of these changes closely mirrored the subjective intensity of DMT’s effects. A novel *delta/theta* rhythmicity emerged during the powerful ‘breakthrough’ period - characterized by complex visionary experiences. Further work is now needed to more closely scrutinize this example of ‘apparent order’ amidst the background of disorder - that is a more recognized feature of the psychedelic state^13,14^. The present study’s findings significantly advance our understanding of the brain basis of one of the most unusual and intense altered states of consciousness known – previously likened to dreaming^40,53^ and the near-death experience^54^. By observing what is lost and gained when consciousness transitions in extreme ways, psychedelic neuroscience promises to enrich our knowledge and appreciation of mind-brain relationships in the broadest range of contexts, while inspiring as yet untold applications.

## Methods

### Participants and experimental procedure

Thirteen healthy participants (6 female, 7 male, mean age, 34.4, SD, 9.1 years) were recruited and provided written informed consent for participation in the study, which was approved by the National Research Ethics (NRES) Committee London – Brent and the Health Research Authority. This study was conducted under the guidelines of the revised Declaration of Helsinski (2000), the International Committee on Harmonisation Good Clinical Practices guidelines, and the National Health Service Research Governance Framework. Imperial College London sponsored the research, which was conducted under a Home Office license for research with Schedule 1 drugs.

Physical and mental health screening consisted in routine physical examination, electrocardiogram, blood pressure and pulse, routine blood tests, as well as a psychiatric interview conducted by a medic. Main exclusion criteria consisted in <18 years of age, having no previous experience with a psychedelic/hallucinogenic drug, personal history of diagnosed psychiatric illness, immediate family history of psychotic disorder, excessive use of alcohol (>40 units per week) and blood or needle phobia. A urine test for drugs of abuse and pregnancy (where applicable) and a breathalyser test were conducted on each study day prior to drug administration.

Participants were asked to attend 2 experimental sessions at the National Institute of Health Research (NIHR) Imperial Clinical Research Facility (CRF). Participants were asked to rest at a semi-supine position with their eyes closed throughout the duration of the experiment and eye shades were placed to promote this. EEG data was then collected from one minute prior to drug administration and placebo up to 20 minutes after. Due to limited data on IV DMT in humans, different doses were given to participants in order to proceed safely in obtaining an adequate dosage of DMT for the purposes of brain imaging. Each participant received one of four doses of DMT fumarate intravenously (three received 7 mg, four received 14 mg, one received 18 mg and five received 20 mg) in a 2 ml sterile saline solution over 30 seconds, which was then flushed with 5 ml of saline over 15 seconds. Administration of placebo (2 ml sterile saline) followed the same procedure as previous studies using intravenous administration of DMT^55,56^. In order to ensure familiarity with the research environment and study team a fixed-order, single-blind design was employed (placebo on the first visit and DMT on the second, which took place a week later). A fixed-order design was used as psychedelics have been shown to induce lasting psychological changes^52^.

Following administration, blood samples were taken at selected timepoints (which were consistent across DMT and placebo sessions) via a cannula inserted in participants’ arm in order to determine DMT levels in plasma. Subjective effects were obtained by asking for intensity ratings every minute in an online fashion (i.e. in real-time, or ‘while it happens’). Also, participants completed Visual Analogue Scales once the subjective effects had sufficiently subsided and ‘micro-phenomenological” interviews were performed the day after the experience with an independent researcher (see below for details).

A 32-channel Brainproducts EEG system (EasycapMR 32) was used at a sampling rate of 1000 Hz. A 0.1 Hz high-pass filter and a 450 Hz anti-aliasing filter was applied. Additional channels were placed for ECG, EMG and EOG activity, with electrodes placed in the chest, the frontalis and temporalis muscles, as well as placed above and below participants’ left eye.

Blood samples of 10 ml were collected in EDTA tubes prior to and at approximately 2, 5, 12, 20, 33 and 60 minutes after DMT administration. Exact sampling times were recorded. After centrifugation at 2500 g for 10 minutes at 4 °C, plasma was harvested and frozen at −80 °C. Samples were shipped to Gothenburg in dry ice for quantitation of DMT by high-pressure liquid chromatography with tandem masspectrometric detection. In brief, acetonitrile was added to plasma aliquots to precipitate proteins. After centrifugation at 13000 g for 10 minutes, 5 µl of supernatant was injected onto the system.

In order to determine the specific progression of effects of DMT over time an independent researcher conducted interviews one day after the DMT session based on the method described by Petitmengin^26,29^. The Micro-phenomenological Interview is a technique that is thought to be able to reduce subjective biases particularly affecting first-person reports^26,28^. It is tailored to facilitate the relationship between subjective experience and its neurophysiological counterparts^29^. During the interview, the recollection of the progression of the subjective experience induced by DMT was aided by using the online intensity ratings which were verbally given by participants, every minute, during the drug sessions. The validity of the reported content was assessed by the interviewer following the method outlined by Petitmengin *et al*.^26^. Three broad dimensions of experience were found to be common to all of the reports: visual, bodily and emotional/metacognitive dimensions. These dimensions were identified by the interviewer and subsequently, the intensity for each of these dimensions was rated by the same researcher for every minute using a 4-point Likert scale for every minute. These ratings were then used for neurophenomenological correlations. The analysis of these interviews was performed in general accordance with the principles associated with micro-phenomenological interviews in which the temporal progression of different dimensions of participants’ experiences is coded by a researcher, rather than the participant^27^, in accordance with the principle of validating first-person reports by the researcher (i.e. thus constituting a ‘second-person’ approach) as outlined by neuropheneomenology^25^.

### Analysis

EEG data was preprocessed using Fieldtrip toolbox^57^. Data was band-pass filtered at 1–45 Hz and was visually inspected. Data containing gross artefacts (jaw clenches, movement) were removed from further analysis, as well as segments in which ratings of intensity were asked and collected on every minute following drug administration. Independent Component Analysis (ICA) was performed and the components associated to eye movements and muscle activity were removed from the data. Comparable amount of components across placebo (mean = 5.58, SD = 2.01) and DMT (mean = 6.2, SD = 1.66) were removed. Alternative ICA cleaning was performed with less components removed (Placebo mean = 4.25 SD = 0.92; DMT mean = 4.9, SD = 0.87), revealing comparable results. The clean data was then re-referenced to the average of all channels and segmented in trials lasting 3 seconds.

**Figure 7:**
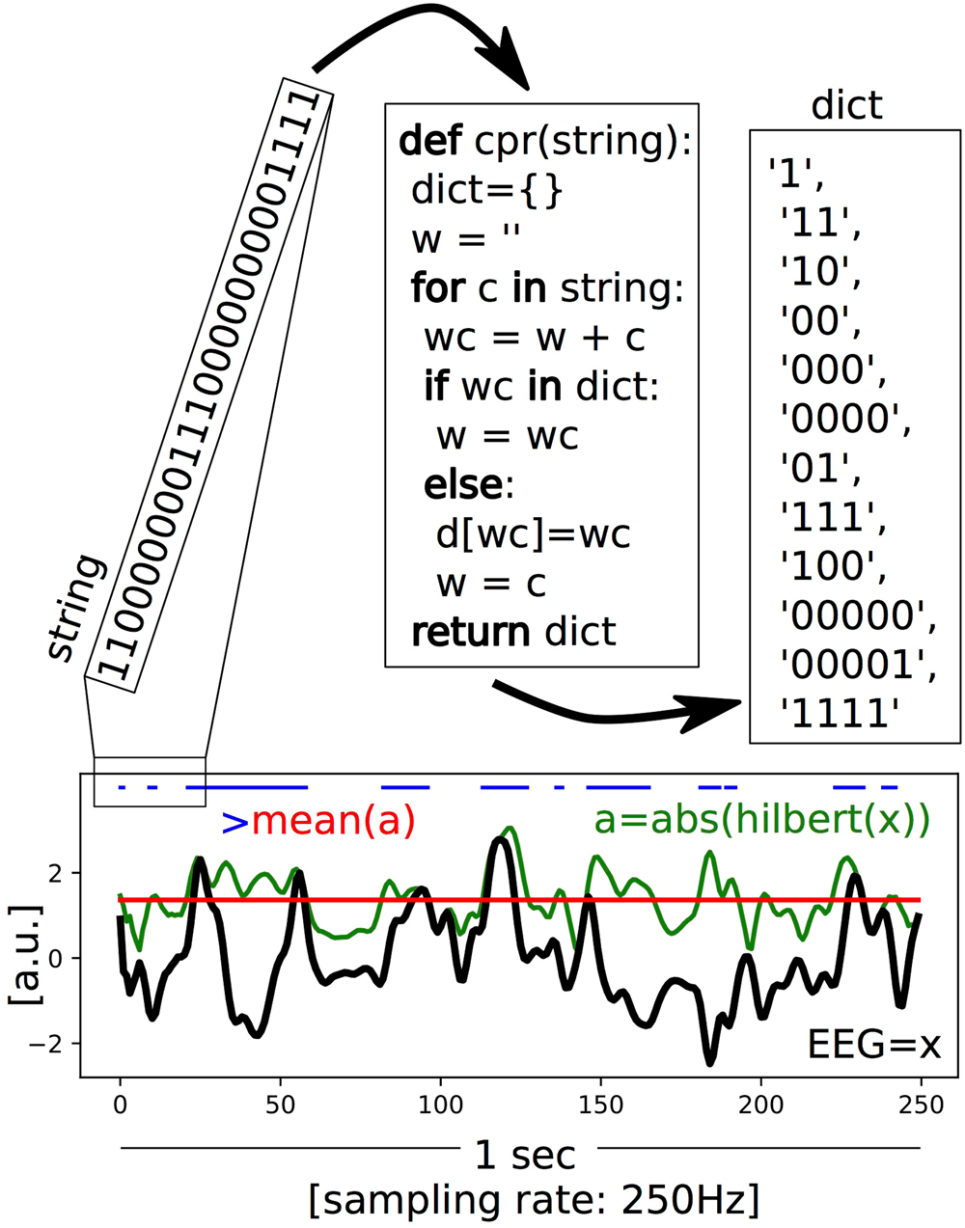
Schematic of the LZs computation. An example EEG signal with a sampling rate of 250 Hz and a length of 1 sec is shown in black (x). The mean (red) of the absolute value of its analytic signal (green, a) is used to binarize the signal (blue). The encoding step of the Lempel-Ziv algorithm is then applied to the first 25 entries of that binarized signal (in this illustration), creating a dictionary of the unique subsequences, which is then normalized by dividing the raw value by those obtained for the same randomly shuffled binary sequence. This provides a value between 0–1 that quantifies the temporal diversity of the EEG signal (LZs).

Spectral and spontaneous signal diversity analysis were performed for 5 minutes-averaged data before and immediately after DMT/Placebo administration (time-averaged results). Additionally, we performed analysis using 1 minute averages throughout the whole 20 minutes of EEG recordings following DMT/Placebo injection (time-sensitive results). Conventional spectral analysis was performed using slepian multitapers with spectral smoothing of +/− 0.5 Hz using the Fieldtrip toolbox^57^. In order to determine the contribution of oscillatory and ‘fractal (1/f) components to spectral power, the signal was decomposed using the Irregularly Resampled AutoSpectral Analysis (IRASA) algorithm, as described by Wen and Liu^16^ (see Supplementary Methods for details). Resulting spectra from both the IRASA algorithm and conventional analysis were divided in the following frequency bands for statistical analysis: *Delta* (1–4 Hz), *theta* (4–8 Hz), *alpha* (8–13 Hz), *beta* (13–30 Hz) and low *gamma* (30–45 Hz). We then computed the spontaneous signal diversity following our previous study^12^, thus obtaining a score for Lempel-Ziv complexity (LZs) and a normalized score for LZs (LZs_N_) in order to ameliorate the effects of spectral power on these results (see Supplementary Methods and Fig. 7 for a schematic on LZs computation).

### Statistical analysis

Paired T-tests were performed at each time point in order to determine the significance of subjective effects determined by real-time intensity ratings and VAS. EEG-related analysis underwent permutation testing of t-statistics to address differences between EEG and placebo. Correlational analyses were performed between (1) Real-time intensity ratings versus EEG measures across time (Fig. 3), (2) Plasma DMT versus EEG measures across time (Fig. 4), (3) Scores extracted from micro-phenomenological interviews and EEG measures across time (Fig. 5) and (4) VAS and EEG measures (Psychometric correlation analysis) (Figs 6 and S3 and S4). Multiple comparison correction was performed using FDR for all measures associated to subjective effects, and cluster randomization analysis was used to control for multiple comparisons of EEG results with an initial cluster-forming threshold of p = 0.05 repeated for 7500 permutations. (see Supplementary Methods for details).

## Supplementary information

Supplementary Information

## Data Availability

The datasets generated during and/or analysed during the current study are available from the corresponding author on reasonable request.
