# Supplementary material for: Neural correlates of the DMT experience assessed with multivariate EEG
Source: Sci Rep. 2019 Nov 19;9:16324. doi: 10.1038/s41598-019-51974-4 (PMC6864083; doi:10.1038/s41598-019-51974-4)
Supplement: Supplementary file 1 — Supplementary Information [file 41598_2019_51974_MOESM1_ESM.pdf]

Supplementary Information

Neural correlates of the DMT experience assessed with multivariate EEG

Christopher Timmermann[1,2*], Leor Roseman[1,2], Michael Schartner[3], Raphael Milliere[4], Luke T. J. Williams[1], David Erritzoe[1], Suresh Muthukumaraswamy[5], Michael Ashton[6], Adam Bendrioua[6], Okdeep Kaur[7], Samuel Turton[8], Matthew M Nour[9], Camilla M Day[1], Robert Leech[10], David J. Nutt[1,8] and Robin L. Carhart-Harris[1,11]

[1]Centre for Psychedelic Research, Department of Brain Sciences, Faculty of Medicine, Imperial College London UK
[2]Computational, Cognitive and Clinical Neuroscience Laboratory (C3NL), Department of Medicine, Imperial College London UK
[3]Department of Basic Neurosciences, University of Geneva, Switzerland
[4]Faculty of Philosophy, University of Oxford, Oxford, United Kingdom
[5]School of Pharmacy, The University of Auckland, New Zealand
[6]PKDM Unit, Department of Pharmacology, University of Gothenburg, Gothenburg, Sweden
[7]Imperial Clinical Research Facility, Imperial College London, London, UK
[8]Centre for Psychiatry, Division of Brain Sciences, Department of Medicine, Imperial College London UK
[9]Department of Psychosis Studies, Institute of Psychiatry, Psychology and Neuroscience, King's College London UK,
[10]Department of Neuroimaging, Institute of Psychiatry, Psychology and Neuroscience, King's College London, UK
[11]Department of Psychiatry, University of Oxford

Corresponding author: Christopher Timmermann. 160 Du Cane Rd, Hammermsith Hospital, Imperial College London, W12 0NN, London. +447752585997. E-mail: c.timmermann-slater15@imperial.ac.uk

Supplementary Figures

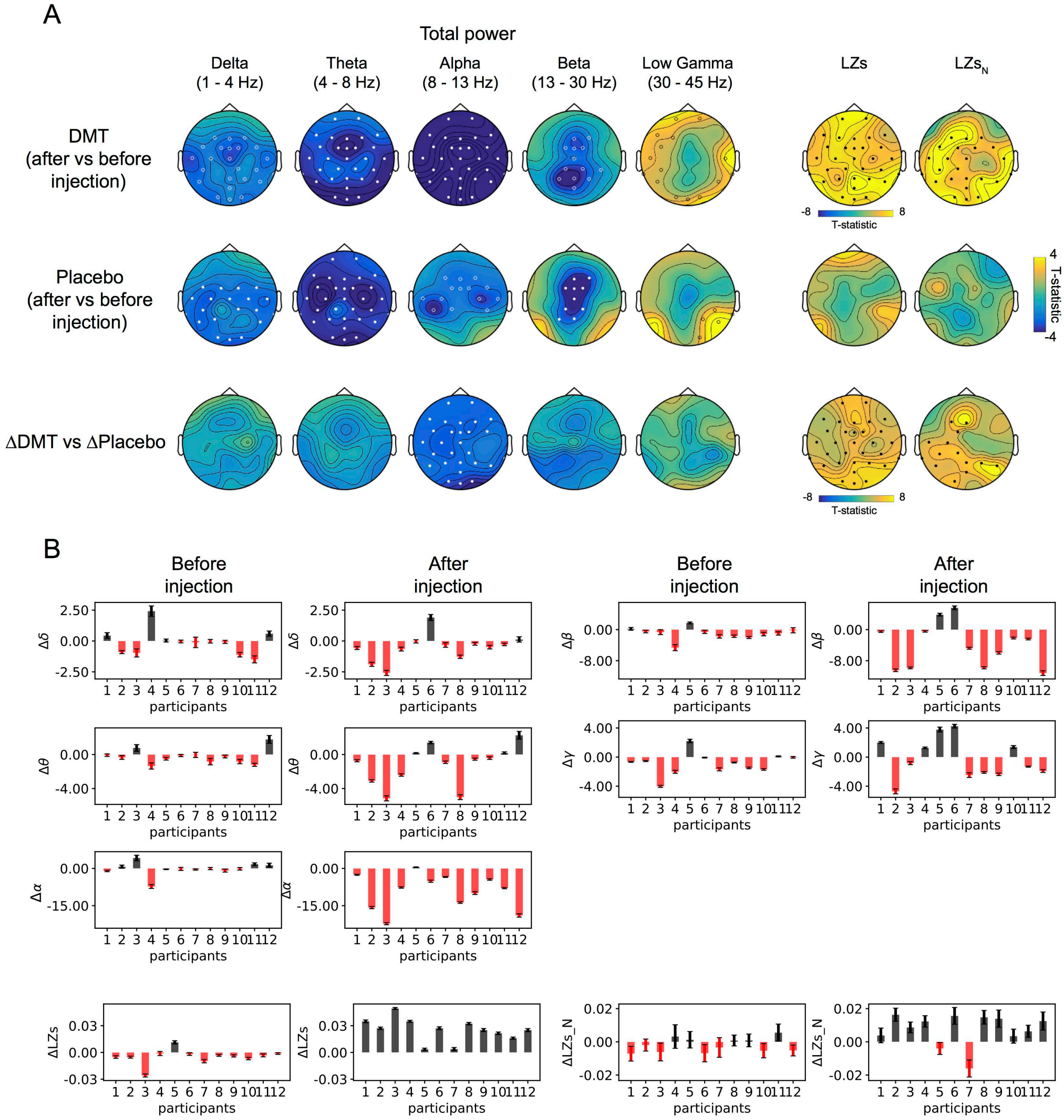

Supplementary Figure S1. Time-averaged EEG results for DMT and placebo separately. (A) Significant decreases in power were found after DMT administration for all frequency bands <30 Hz and increases were found for low *gamma* and spontaneous signal diversity. Results from the placebo condition showed reductions in power for *delta*, *theta*, *alpha* and *beta* bands, while minor increases were seen for low *gamma* and LZs. The contrast of DMT versus placebo while taking into account baseline recordings revealed that DMT had an effect on decreases in *alpha* power, as well as increases in LZs and LZs_N. Filled circles correspond to clusters $p<0.01$ and hollow circles for clusters $p<0.05$, N=12. (B) Differences (DMT minus placebo) for averaged activity across all EEG channels at all frequency bands and signal diversity measures before and after injection (δ = *delta*, θ = *theta*, α = *alpha*, β = *beta*, γ = *gamma*, LZs = Lempel-Ziv complexity, LZs_N = normalized LZs).

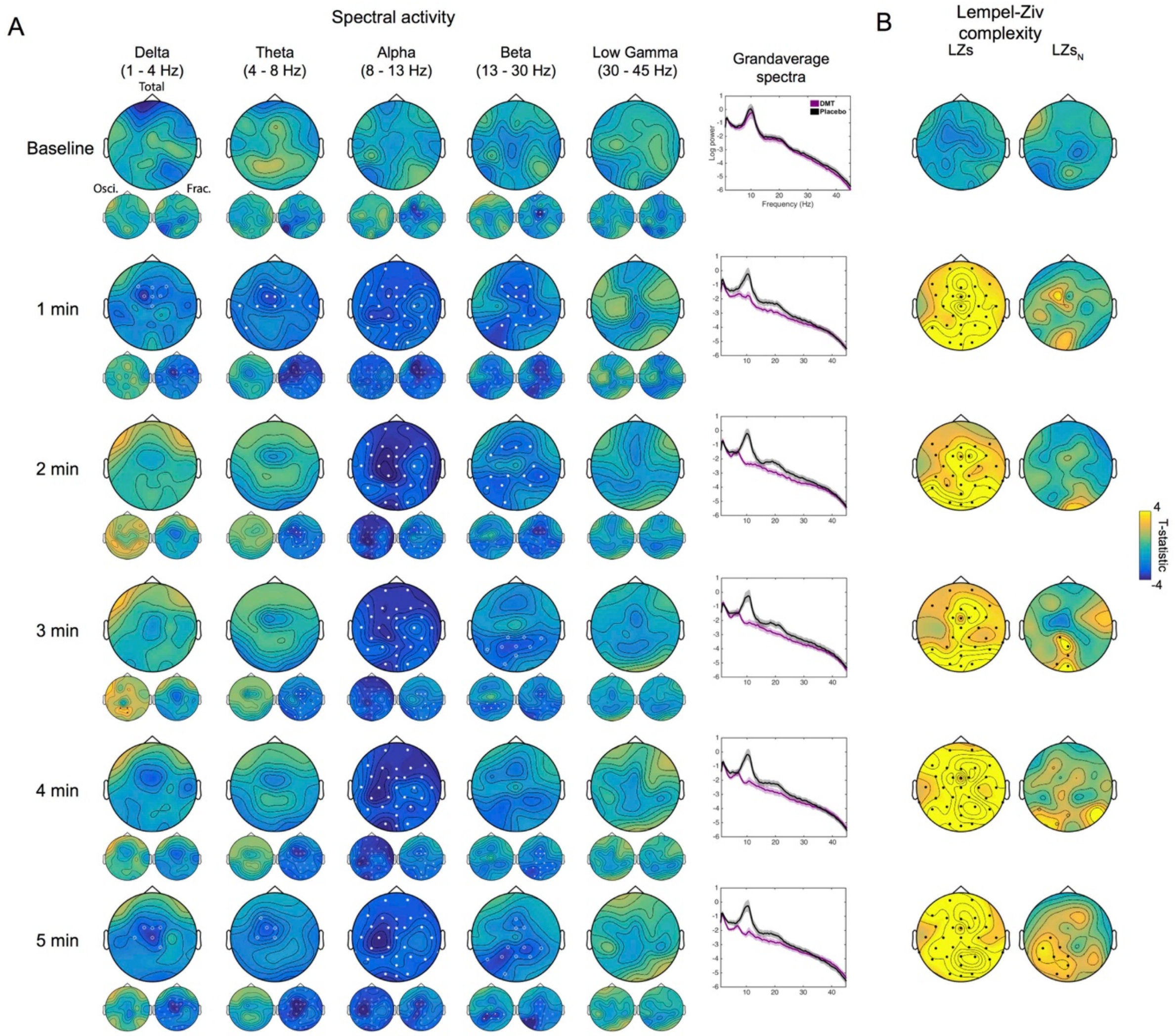

Supplementary Figure S2. Time-sensitive EEG results. (A) Results of the comparison of DMT versus placebo using averaged data for each minute, for a total of the baseline and 5 minutes post-injection for spectral activity and the corresponding grandaverage spectral plots (mean ± SEM). Total, oscillatory and fractal power are displayed. (B) Results using complexity measures LZs and LZs_N. Filled circles correspond to clusters $p<0.01$ and hollow circles for clusters $p<0.05$, N=12. Notice how reductions in lower frequency bands for total power are associated to reductions in power in the fractal (and not oscillatory) component. Also notice the transient increases in *delta* power at minute 3 only for the oscillatory component of the signal.

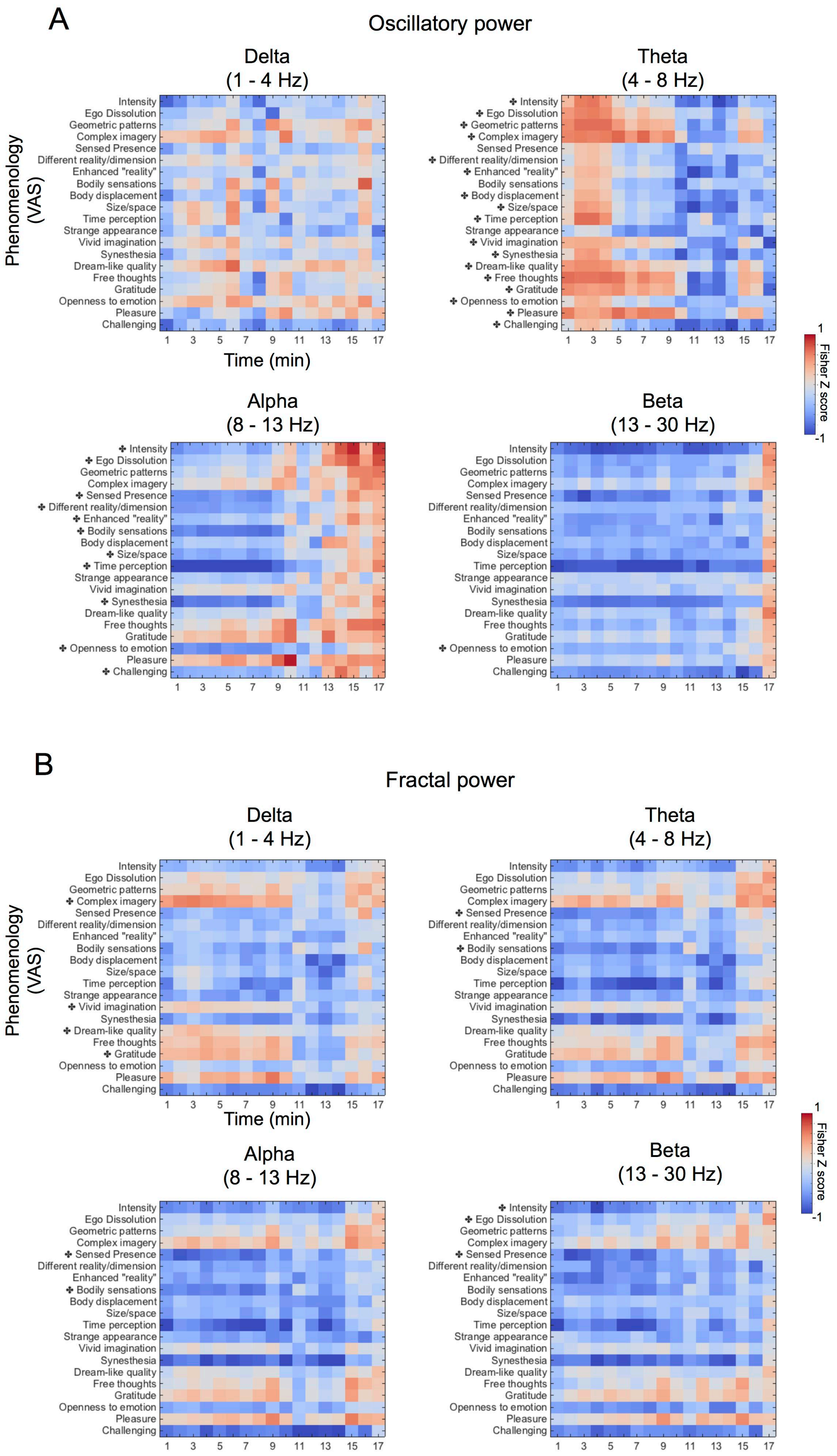

Supplementary Figure S3. Psychometric correlations for oscillatory and fractal power. Normalized correlation coefficient values between VAS items and EEG measures for each minute following DMT administration reveal a significant relationship with mean online ratings of intensity for (A) oscillatory and (B) fractal power. Significant correlations are marked with a cross following Bonferroni-correction for multiple comparisons at $p<0.05$. Notice the predominance of the *theta* band for psychometric correlations using oscillatory power and the reduced relevance of all bands using fractal power. See Fig. 6A for results concerning total power.

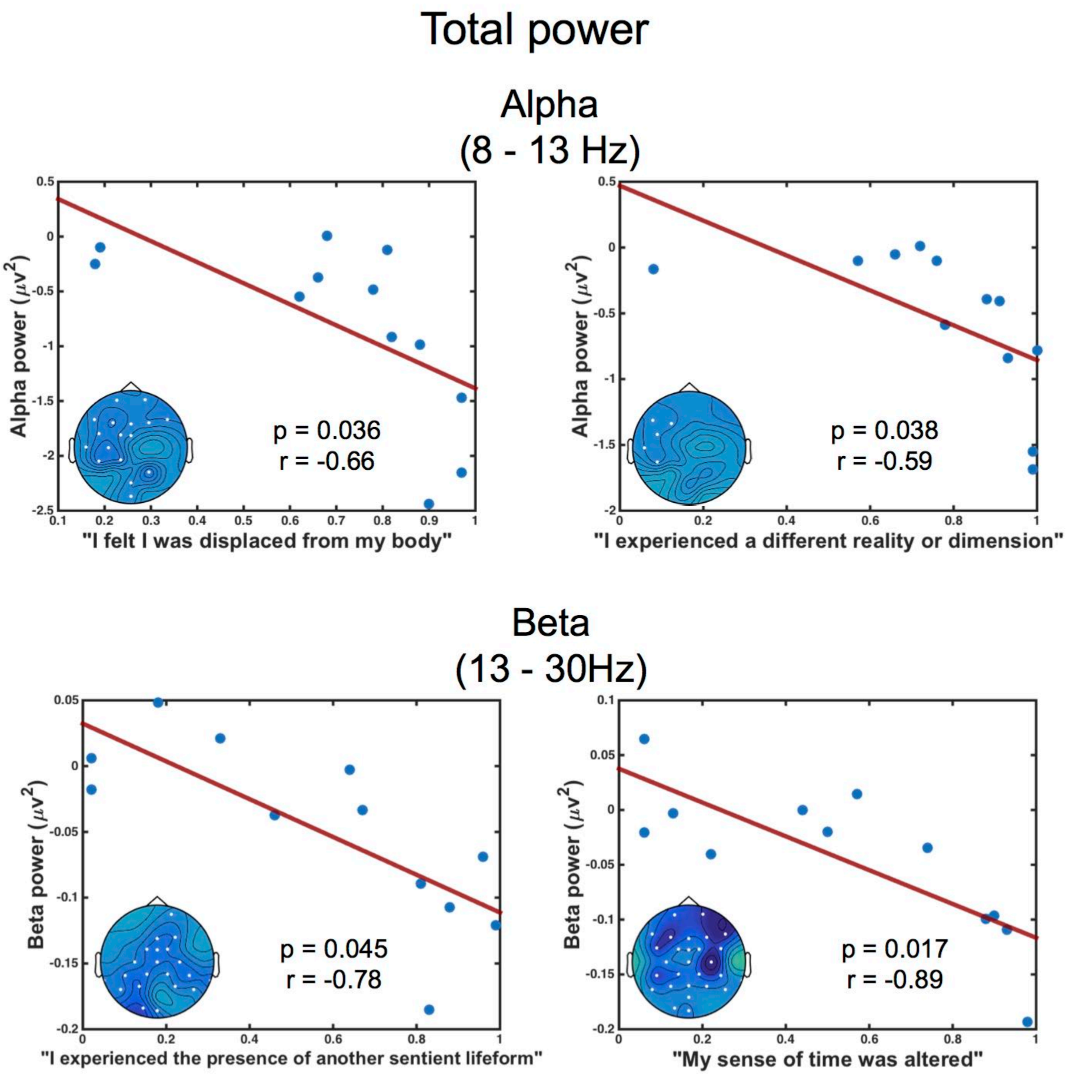

Supplementary Figure S4. Time-averaged (5-minutes) psychometric correlations. Significant correlations between power at *alpha*/*beta* bands (total power for 5 minutes averaged data) and VAS retrospective scores (FDR corrected) (plots display significant channels corresponding to averaged DMT minus placebo activity of total power).

Supplementary Methods

Safety: Ensuring psychological safety is crucial in studies involving psychedelic compounds[1], therefore special attention was given to the physical context and care was placed on the quality of interpersonal interactions between researchers and participants, as these factors are known to influence psychological safety[1,2] and DMT can induce intense emotional effects[3,4]. For this reason, the clinical environment was carefully decorated, maintaining consistency across participants and DMT/placebo sessions. Mood lights, candles and sober decoration was used. When possible medical equipment was covered with blank sheets in order to prevent associations with a sterile, medical context. Additionally, low-volume music was used during the sessions to further provide an experience of comfort and safety and a simple relaxation technique (body scan) was used prior to the administration of DMT and placebo.

Subjective effects: A measure of subjective effects, related to the intensity of drug effects was obtained by asking to rate the intensity of subjective effects from 0 to 10 on every passing minute following drug administration for a total of 20 minutes after injection. Anchors related to the extreme of the scale were explained to participants with a rating of 0 corresponding to "no drug effects" and 10 to "the strongest drug effects imaginable". Immediately after the effects of DMT subsided and participants were able to do so, (or at ~30 minutes following placebo administration) Visual Analogue Scales (VAS) were completed by participants. Participants were asked to rate each item in relation to the moment in which peak effects of the drug were subjectively felt. The items used were based on effects seen in previous research conducted with other psychedelics[5–7] as well as some of the reported characteristic effects of DMT[8–10].

IRASA: In order to determine the contribution of oscillatory and 'fractal (1/f) components to spectral power, the signal was decomposed using the method described by Wen and Liu[11]. The Irregularly Resampled AutoSpectral Analysis (IRASA) algorithm is able to obtain an approximate estimate of the power spectra of the oscillatory component of the signal as follows: Each epoch is segmented in overlapping subepochs which comprise 90% of the signal. The Fast-Fourier Transform (FFT), tapered

with a Hanning window is applied obtaining the power spectra for each of the subepochs. Each of the subepochs are then resampled (upsampled and downsampled) by factors of h and 1/h (we used a range of h going from 1.1 and 2.9 using an increment of 0.05 as in Muthukumaraswamy et al.[12]). The power spectra are obtained using the same method as for the non-resampled segments. The geometric mean across the resampled power spectra for each of their corresponding subepochs is obtained which results in an estimation of the "fractal" component. The estimated power of the fractal and original epochs are averaged across all subepochs and the average spectrum of the fractal component is subtracted from the power spectrum of the original signal, which yields an estimate of the oscillatory component for each epoch.

Spontaneous signal diversity analysis: We computed Lempel-Ziv complexity following our previous study[13] (python code https://github.com/mschart/SignalDiversity). The instantaneous amplitude (obtained via Hilbert transform) of each source channel is binarised using its mean over observations as a threshold, i.e. the continuous signal of each source channel is transformed into a string of 3000 binary digits (for our case of 3sec segments at 1000 Hz sampling rate), resulting in a matrix with binary entries with a row for each channel and a column for each observation. To assess the signal diversity across observations, the encoding step of the Lempel-Ziv 1978 (LZ78) compression algorithm (implemented by adapting open source code) is applied to the binary string of each channel. The LZ78 algorithm divides the string into non-overlapping and unique binary substrings. The more diverse the binary string, the more substrings will be listed, with a minimal number of substrings for a sequence of 0s (1s) only. The total number of these substrings for a given channel is what we call Lempel-Ziv complexity (LZs), capturing temporal signal diversity of single channels. We normalize LZs by dividing the raw value by the value obtained for the same binary input sequence randomly shuffled. Since the value of LZs for a binary sequence of fixed length is maximal if the sequence is entirely random, the normalized values indicate the level of signal diversity on a scale from 0 to 1.

In order to test if changes in the diversity measures in the drug vs placebo contrast can be completely explained away by changes in the overall power spectrum, we employed the following control

procedure. As in Schartner et al.[13], we assume that effects of the spectral profile on LZs' behavior are multiplicative and thus we divided the LZs score for data by the LZs score of the data after we phase-randomised it. Phase randomisation was performed by first applying a discrete Fourier transform, then randomising phases of the frequencies while keeping their amplitudes fixed, and then applying the inverse Fourier transform (i.e. only the relative temporal positions [phases] of the Fourier sinusoids, whose superposition equals the signal, are randomly changed). We denote Lempel Ziv complexity measures normalised by average scores for phase-randomised data as LZs_N. All computations were implemented in python.

Statistical analysis of EEG results: For analysis on time-averaged spectral activity (on the frequency bands reported before) and spontaneous signal diversity, five minute segments of EEG activity before, and 5 minutes and 30 seconds following the end of drug injection were used for each condition. An additional thirty seconds were used for the post-injection data to account for the segments discarded related to the verbal ratings participants were asked on every minute after injection (interruptions lasting ~6 s). Permutation testing of t-statistics were used to address differences between DMT and placebo for time-averaged results. Cluster randomization analysis was used to control for multiple comparisons with an initial cluster-forming threshold of $p = 0.05$ repeated for 7500 permutations. Additionally, peak frequency in the 4-45 Hz range was calculated for DMT and placebo for the average of all channels and a paired t test was performed. Additionally, DMT vs placebo analyses were performed for averaged EEG activity at each minute following administration (see *Time-sensitive EEG results* in the main article text). Two-tailed hypothesis testing were made for all analyses. The channel displaying the absolute maximum t value is reported and the cluster p value is reported.

Statistical analysis of subjective vs EEG effects across time: For the analyses exploring the relationship between EEG measures and real-time intensity a two-step approach was performed. At the first-level an r value was obtained by correlating the intensity ratings (for the significant 1-17 minutes period after injection) and EEG data corresponding to minute-by-minute averages obtained

following DMT administration. This procedure was repeated for each electrode resulting in an r value per electrode for each participant. Statistical significance was then established at the second level by using permutation testing of t-statistics (as described in the section above) comparing the resulting r values obtained in the previous step against a null condition (in which the r value was replaced by a ‘0’). The same cluster randomization analysis performed in averaged EEG data was used here to control for multiple comparisons. Two-tailed hypothesis testing were made for all analyses. The channel displaying the absolute maximum t value is and the cluster p value are reported.

Statistical analysis of plasma DMT vs EEG analysis*:* The relationship between plasma levels of DMT and EEG activity was obtained using the same two-step method described above, replacing intensity ratings by DMT concentrations in plasma. The segments of EEG data used in this case were one minute averages which were selected in time by keeping a temporal correspondence to each moment in which participants’ blood was drawn.

Statistical analysis of neurophenomenology: The relationship between scores extracted from micro-phenomenological interviews (see Methods in the main text for details) and EEG measures was determined using the same method as real-time intensity, with the difference that ratings of real-time intensity were replaced by ratings for each of the dimensions of experience (visual, bodily and emotional/metacognitive) across time in three separate analyses. Similarly, analyses were limited to the significant 1-17 minute period post-DMT administration.

Statistical analysis of psychometric correlations: In order to address the temporal relationship between subjective effects as determined by VAS and EEG data, post-hoc exploratory correlation analysis was performed using minute–by-minute averages (across all channels) of EEG measures showing significance in previous analyses (*delta, theta, alpha, beta,* LZs and LZs_N) and all VAS items. In order to normalize individual differences, we used DMT minus placebo data at each corresponding minute (previous time-resolved analyses do not require this step as correlation analysis is done at the single-subject level). These analyses were performed for each minute in the significant 1-17 minutes

range post-administration. The resulting values (an r coefficient at each minute and for each VAS item) were normalized using the Fisher's Z transform and subsequently each VAS item was correlated with the average online intensity obtained across participants. In order to increase the specificity of these findings (due to the large amount of significant results) we performed stringent bonferroni-correction across all results in order to determine the amount of VAS items relating to each EEG measure.

Time-averaged psychometric correlations: In addition to minute-by-minute correlation analysis we performed more conventional (but less-sensitive) correlational analysis using 5-minutes averaged EEG data. Correlation analysis were performed between differences between drug conditions for each VAS item and changes seen in *alpha* and *beta* band following injection of DMT and placebo, as well as LZs as these were the measures showing the most consistent effects of DMT, explaining separate contributions of the EEG signal. Correlations were performed on the VAS items related to the characteristic effects specific to DMT[4,9] (i.e. the VAS items "I experienced a different reality or dimension" and "I experienced the presence of another sentient lifeform") and ratings of ego-dissolution. FDR correction was applied and two-tailed hypothesis testing was made.

Supplementary References

1. Johnson, M., Richards, W. & Griffiths, R. Human hallucinogen research: guidelines for safety. *J. Psychopharmacol.* **22**, 603–620 (2008).
2. Studerus, E., Gamma, A., Kometer, M. & Vollenweider, F. X. Prediction of psilocybin response in healthy volunteers. *PLoS One* **7**, (2012).
3. Strassman, R. J. Human psychopharmacology of N,N-dimethyltryptamine. *Behav. Brain Res.* **73**, 121–124 (1995).
4. Strassman, R. *DMT: The spirit molecule*. (Park Street Press, 2001).
5. Carhart-Harris, R. L. *et al.* Neural correlates of the psychedelic state as determined by fMRI studies with psilocybin. *Proc. Natl. Acad. Sci.* **109**, 2138–2143 (2012).
6. Carhart-harris, R. L. *et al.* Neural correlates of the LSD experience revealed by multimodal neuroimaging. *Proc. Natl. Acad. Sci.* **113**, 4853–4858 (2016).
7. Muthukumaraswamy, S. D. *et al.* Broadband cortical desynchronization underlies the human psychedelic state. *J. Neurosci.* **33**, 15171–15183 (2013).
8. Sai-Halász, A., Brunecker, G. & Szára, S. Dimethyl-tryptamin : ein neues Psychoticum. *Psychiatr. Neurol. (Basel).* **135**, 258–301 (1958).
9. Strassman, R. J., Qualls, C. R., Uhlenhuth, E. H. & Kellner, R. Dose-Response Study of N,N-Dimethyltryptamine In Humans. II. Subjective effects and preliminary results of a new rating scale. *Arch. Gen. Psychiatry* **51**, 98–108 (1994).
10. Gouzoulis-Mayfrank, E. *et al.* Psychological effects of (S)-ketamine and N,N-dimethyltryptamine (DMT): a double-blind, cross-over study in healthy volunteers. *Pharmacopsychiatry* **38**, 301–311 (2005).
11. Wen, H. & Liu, Z. Separating Fractal and Oscillatory Components in the Power Spectrum of Neurophysiological Signal. *Brain Topogr.* **29**, 13–26 (2016).
12. Muthukumaraswamy, S. D. & Liley, D. T. 1/f electrophysiological spectra in resting and drug-induced states can be explained by the dynamics of multiple oscillatory relaxation processes. *Neuroimage* **179**, 582–595 (2018).
13. Schartner, M. M., Carhart-Harris, R. L., Barrett, A. B., Seth, A. K. & Muthukumaraswamy, S. D. Increased spontaneous MEG signal diversity for psychoactive doses of ketamine, LSD and psilocybin. *Sci. Rep.* **7**, 46421 (2017).
